# Safety outcomes of teclistamab accelerated dose escalation

**DOI:** 10.1177/10781552241268429

**Published:** 2024-08-01

**Authors:** Yumena Kawasaki, Aaron Paul Steele, Aaron Rosenberg, Julie Guglielmo

**Affiliations:** 1 Department of Pharmacy, 8789 University of California Davis Medical Center, Sacramento, CA, USA; 2 Comprehensive Cancer Center, Division of Malignant Hematology, Cellular Therapy & Transplantation, 8789 University of California Davis Medical Center, Sacramento, CA, USA

**Keywords:** Multiple myeloma, teclistamab, B-cell maturation antigen (BCMA), cytokine release syndrome, immune effector cell-associated neurotoxicity syndrome (ICANS)

## Abstract

**Introduction:**

Teclistamab, a bispecific T-cell engaging antibody targeting B-cell maturation antigen (BCMA), is indicated for the treatment of relapsed or refractory multiple myeloma after at least four lines of therapy. It has boxed warnings for life threatening cytokine release syndrome (CRS) and immune effector cell-associated neurotoxicity syndrome (ICANS). To mitigate these risks, teclistamab is initiated using step-up doses. This article examines safety event rates following the implementation of a 2-day separation between step-up doses at one institution to streamline patient care.

**Methods:**

This was a retrospective, single-center study encompassing all patients who received teclistamab within a 1-year period. The primary endpoint was the overall incidence of CRS and ICANS. Secondary endpoints included hospital length of stay, hematological toxicities, infection rates, among other adverse events.

**Results:**

A total of 27 patients were included in the analysis and stratified into accelerated (days 1,3,5) or standard (days 1,4,7) dosing groups. CRS occurred in 48% (11) of patients for the accelerated dosing and 50% (2) for the standard dosing group. ICANS was seen in 17% (4) of patients in the accelerated dosing group and none in the standard dosing group. Average length of stay in the accelerated dose was 7.6 days versus 9.2 days in the standard dose group.

**Conclusion:**

Accelerated dose escalation of teclistamab yielded safety event rates comparable to those in the literature. These findings may support outpatient administration for teclistamab. Accelerated dose escalation strategy allowed for the optimization of hospitalization and resources.

## Background

Multiple myeloma is a malignancy of the plasma B-cells with an abnormal increase in monoclonal immunoglobulins. Multiple myeloma is often characterized by its hallmark symptoms of hypercalcemia, anemia, renal failure, and bone lesions along with ≥10% clonal bone marrow plasma cells. Median age of diagnosis is 70 years and accounts for about 1.8% of new cases in the United States each year.^1^

Standard of care treatment typically includes a three-drug regimen with a proteasome inhibitor (PI), immunomodulatory agent (IMiD), and dexamethasone followed by autologous transplantation and maintenance therapy.^2^ Despite the advent of stem cell transplant, recurrence and relapse rates remain high. Several new therapies have been approved in recent years, including chimeric antigen receptor (CAR) T-cell therapy and bispecific antibody therapy (BsAbs) which provide more treatment options in this difficult to treat population.^3^

Teclistamab-cqyv (Tecvayli) is a novel bispecific T-cell engaging antibody used in the treatment of refractory or relapsed multiple myeloma and for patients who have used ≥4 lines of prior therapy including an IMiD, a PI, and an anti-CD38 antibody.^4^ Teclistamab targets CD3 on T-cells and B-cell maturation antigen (BCMA) on myeloma cells to trigger cell lysis through T-cell mediated cytotoxicity. Teclistamab received FDA approval on October 25, 2022 under the accelerated approval pathway based on the MajesTEC-1 trial where 165 patients who had previously received at least three lines of therapy were administered teclistamab.^5^ At a median follow-up of 14.1 months, teclistamab achieved an overall response rate (ORR) of 63% with 39.4% having complete response (CR). Median duration of response was 18.4 months and median duration of progression-free response was 11.3 months.

Teclistamab is associated with boxed warnings for life threatening or fatal cytokine release syndrome (CRS) and neurologic toxicity, including immune effector cell-associated neurotoxicity (ICANS) requiring Risk Evaluation and Mitigation Strategy (REMS) medication monitoring. CRS is the systemic release of pro-inflammatory cytokines triggered by a variety of factors including monoclonal antibodies causing symptoms ranging from mild, such as fever and chills, to severe, such as organ failure.^6^ ICANS is inflammation of the central nervous system (CNS) resulting in neurological symptoms such as confusion, headache, attention deficits, word finding difficulties, and even seizures.^7^ In the MajesTEC-1 trial, CRS was observed in 72.1% of patients and most events were limited to grade 1 or 2 without any treatment discontinuations. ICANS was observed in 3% of patients and all events were limited to grade 1 or 2.

Teclistamab is a subcutaneous (SQ) injection with recommendations for inpatient monitoring during the initial dose-ramp up phase to reach the maintenance dose. The cycle duration is 28 days and patients may continue until disease progression or unacceptable toxicity. Premedication is required with all step-up doses and with the first treatment dose to reduce the risk and severity of CRS. The standard step-up dosing for teclistamab is for step-up dose one (0.03 mg/kg) to be administered on cycle one day one, the second step-up dose (0.6 mg/kg) on day four, and the first full treatment dose (1.5 mg/kg) on day seven with weekly maintenance doses to follow. Hospitalization is recommended for 48 hours following administration of all doses in the step-up administration schedule. The package insert allows for the second step-up dose to be given 2 to 4 days after the first dose and the first treatment dose to be administered 2 to 4 days after the second step-up dose. The University of California (UC) Davis Health system adopted a 2-day dose escalation policy for teclistamab to streamline patient care and hospitalization admissions. No standardization exists regarding the safety and length of stay on accelerated dose escalation and no data has been published regarding the use of an accelerated dose in comparison to a standard dose strategy. The objective of this study was to characterize the safety events surrounding the accelerated dose escalation of teclistamab.

## Methods

This was a retrospective, single-center, exploratory study. Patients were identified from the electronic medical record (EMR) using a data analytics tool for all teclistamab administrations in the time period of December 2022 to December 2023. Patients were excluded from analysis if they did not receive the second step-up dose of teclistamab. The primary endpoint was the overall incidence of CRS and ICANS using the American Society for Transplantation and Cellular Therapy (ASTCT) 2019 grading scale. Secondary endpoints included length of stay defined as a continuous period of hospitalization during all teclistamab step-up dosing administrations, as well as other safety parameters such as hematological toxicities (neutropenia, anemia, thrombocytopenia), infection rates, hepatotoxicity, diarrhea, and injection site reactions. Descriptive statistics were used to describe all outcomes in this study.

## Results

**Figure 1.**
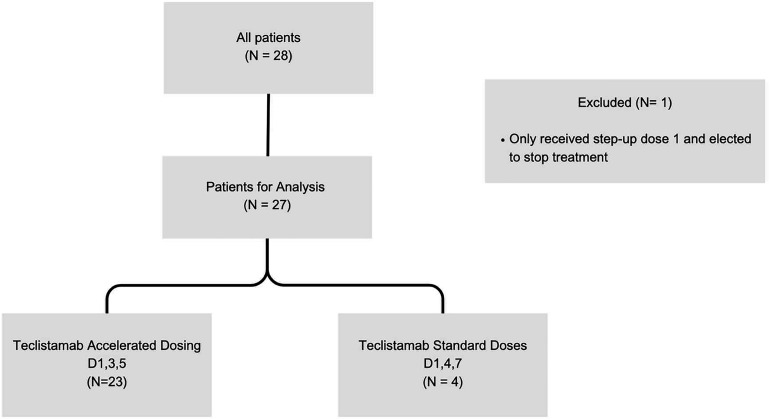
Consort diagram.

A total of 28 patients were identified as receiving teclistamab in the past year; one patient was excluded due to electing to stop treatment after receiving the first step-up dose leaving 27 patients for analysis (Figure 1). Out of the 27 total patients, 23 (85%) patients were scheduled to have the accelerated (day 1, 3, 5) step-up dosing schedule while 4 (17%) patients were scheduled to receive the standard dosing (day 1, 4, 7). The 4 patients in the standard dosing group were from the institution's first few teclistamab administrations before the standardized accelerated dose strategy was implemented.

Baseline characteristics in the overall population included a median age of 69 years, five median prior lines of therapy, previous stem cell transplant in 68% of patients, and extramedullary disease seen in 30% of patients (Table 1).

**Table 1.** Baseline characteristics.

Characteristic	Teclistamab D1,3,5 (*n* = 23)	Teclistamab D1,4,7 (*n* = 4)	Teclistamab composite (*n* = 27)
Age, median (years)	69	64	69
Male sex, no. (%)	14 (61)	1 (25)	15 (56)
Median lines of therapy	5	5	5
Caucasian, no. (%)	16 (69)	2 (50)	18 (67)
Other race, no. (%)	7 (30)	2 (50)	9 (33)
CKD, no. (%)	7 (27)	0 (0)	7 (27)
Extramedullary disease, no. (%)	6 (26)	2 (50)	8 (30)
ECOG 1, no. (%)	12 (52)	1 (25)	13 (48)
Previous IMID, no. (%)	23 (100)	4 (100)	27 (100)
Previous PI, no. (%)	22 (95)	4 (100)	26 (96)
Previous anti-CD38, no. (%)	23 (100)	4 (100)	27 (100)
Previous HSCT, no. (%)	15 (65)	3 (75)	18 (68)
Kappa light chains, no. (%)	14 (61)	3 (75)	16 (59)

## Primary outcome

### CRS

**Figure 2.**
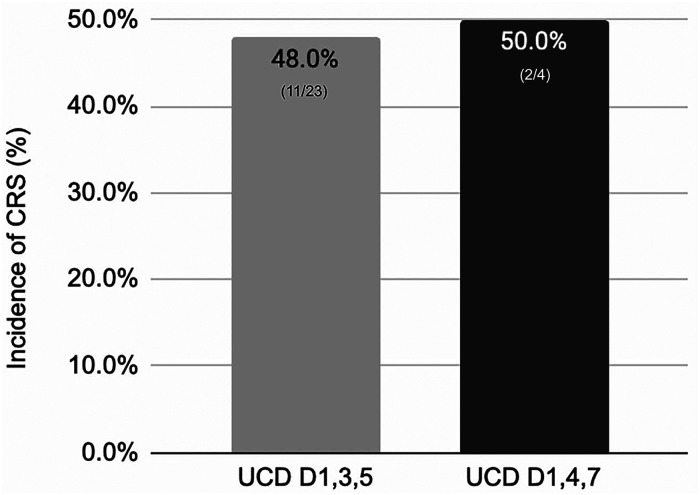
Overall CRS rates.

The overall incidence of CRS for the accelerated dose group was 48% (11) and 50% (2) for the standard dose group (Figure 1). Grade 1 CRS was seen in 7 (30%) patients, grade 2 in 4 (17%) patients, with no grade 3 or higher events reported. CRS occurred after step-up dose 1 in 4 patients, after step-up dose 2 in 1 patient, and after the first full treatment dose in 6 patients. Tocilizumab was used in 35% (8) of patients. Steroid use was seen in 1 (4%) patient who had concurrent ICANS. Out of the 23 patients intended to receive the accelerated dosing schedule, 15 (65%) received the doses on time while 8 (35%) had dose delays due to CRS or ICANS events. From the standard dose group, CRS was seen in a total of 2 patients with 1 event being grade 1 (25%) and 1 event being grade 2 (25%). Tocilizumab use was seen in 1 (25%) patient. No recurrent CRS or treatment discontinuation was seen in either group (Figure 2).

### ICANS

ICANS was seen in 4 patients (17%) out of the accelerated dosing group: grade 1 (25%), grade 2 (25%), grade 3 (25%), and grade 4 (25%). Permanent discontinuation due to ICANS was seen in 1 patient. Steroid use was seen in 3 (75%) patients for ICANS management. Dose delays due to ICANS symptoms were seen in 3 patients. Three of the patients had confounding factors for neurotoxicity such as active infection, high ammonium levels due to uncontrolled myeloma, and history of hospital acquired delirium. ICANS was not seen in the standard dosing group.

## Secondary outcomes

### Length of stay

**Figure 3.**
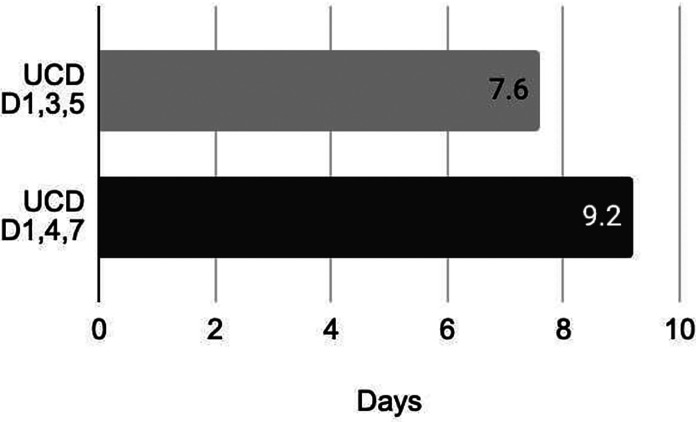
Average length of stay between accelerated dosing and standard dosing group.

Length of stay in the accelerated dosing group was an average of 7.6 and 9.2 days in the standard dosing group (Figure 3). A majority (73%) of the accelerated dose population received doses on schedule without dose delay from CRS/ICANS. Longer hospitalization due to CRS/ICANS was seen in 8 (35%) of patients in the accelerated dosing group and 1 (25%) in the standard dosing group. Extended hospitalization due to a reason other than CRS was seen in 3 (13%) in the accelerated dosing group and 1 (25%) in the standard dosing group with the common reason being infections.

### Adverse events

The most common adverse event rates seen in the accelerated dosing group included infections (42%), diarrhea (39%), and neutropenia (22%) with (17%) experiencing grade 3 neutropenia. Other adverse events are as follows: leukopenia (17%), injection site reactions (13%), thrombocytopenia (4%), hepatotoxicity (1%), and anemia (0%). In the standard dose group neutropenia (100%) with 75% experiencing grade 3 neutropenia, leukopenia (25%), infections (25%) were the most common and thrombocytopenia, diarrhea, hepatotoxicity, anemia and injection site reactions were not seen (0%) (Table 2).

**Table 2.** Secondary outcomes.

Adverse events	Teclistamab D1,3,5 (*n* = 23)	Teclistamab D1,4,7 (*n* = 4)	Teclistamab composite (*n* = 27)
Neutropenia, no. (%)	5 (22)	4 (100)	8 (30)
Thrombocytopenia, no. (%)	1 (4)	0 (0)	1 (3)
Leukopenia, no. (%)	4 (17)	1 (25)	5 (19)
Infections, no. (%)	10 (43)	1 (25)	11 (41)
Diarrhea, no. (%)	9 (39)	0 (0)	9 (33)
Hepatotoxicity, no. (%)	1 (4)	0 (0)	1 (3)
Injection site reactions, no. (%)	2 (13)	0 (0)	2 (7)

## Discussion

To our knowledge, this is the first retrospective study examining CRS and ICANS rates with teclistamab accelerated dose escalation. Overall we observed comparable rates of CRS and ICANS with those in the literature. Baseline characteristics were comparable to that of the real-world population with the exception of extramedullary disease. Our population had higher rates of extramedullary disease (26%) compared to the MajesTEC-1 (17%) trial indicating a population with higher disease burden.

Overall CRS rates were relatively lower than reported in the literature in both cohorts. For the accelerated dose group the rate of CRS was 48% (11) and 50% (2) in the standard dose group which are both less than what was seen in the MajesTEC-1 trial (72%). Tocilizumab use was lower (35%) in our population than in the trial (36.4%). This lower use rate remained true despite an institutional policy for teclistamab allowing earlier intervention with tocilizumab for unresolved grade 1 CRS beyond 24 hours. We examined other real-world population trials of teclistamab administration to contextualize the results of this study. The real-world population trials have variable dose administration strategies ranging from standard dosing to a mix of dosing strategies. The overall CRS rates across these trials ranged from 36% to 64%.^8–10^ The results of this study reaffirm the safety of current practices at our institution of an accelerated dose strategy for BsAb administration.

The ICANS rate in the accelerated group was 17% (*n* = 4) compared to none (0%) in the standard dose group (*n* = 0). ICANS rates in the accelerated group (17%) in our study seem to be higher than MajesTEC-1 (3%), although 3 out of 4 patients in this group had potential confounding factors for neurotoxicity. Of note the ICANS rate for teclistamab in the package insert is 6% indicating a discrepancy between the package insert and the trial data indicating the real-world population ICANS rates may be higher. This theory is further supported by various real-world population studies showing a range in ICANS rates between 7% and 15%.^8–10^ Reasons for high ICANS rates in our study may be due to the limited population (*n* = 4/23), higher extramedullary disease burden, and confounding factors for neurotoxicity. More data is required to identify differences in safety event rates between accelerated versus standard dosing.

Length of stay was optimized with the accelerated dosing schedule. The accelerated dosing schedule resulted in an average 1-day reduction in inpatient admission, equating to approximately $10,071 in savings per patient per day.

The most common secondary adverse events seen for the accelerated dose group included neutropenia (22%) and infections (43%); these rates were less than what was observed in the MajesTEC-1 trial (neutropenia 71%; infections 76%).

Limitations of this study include limited sample size, retrospective design, and a lack of a direct comparison arm. With the limited sample size, this study was not powered to detect statistically significant differences. A larger patient population is necessary to determine safety event rates between accelerated and standard dosing of teclistamab. Determining patient's risks for CRS and ICANS may help triage inpatient admissions for step-up dosing and stratify patients who may be able to receive the medications in the outpatient setting.

### Clinical applicability

As an institution, UC Davis Health is working towards administration of the step-up dosing of BsAbs in the outpatient setting in the future. With this transition, the goal is to prioritize hospital resources and improve patient experience by minimizing hospitalization. The expected rates of CRS and ICANS found in this study may support the safety of outpatient bispecific step-up dosing. Further evaluation of administration strategies should be considered including: home monitoring strategies, robust patient education, and a CRS and ICANS management and triage process. Some institutions have experimented with teclistamab initiation in the outpatient setting. Per Mayo et al.,^11^ teclistamab was administered on days 1,4,7 while a study^12^ at Fox Chase Cancer center administered it on days 1,3,8; CRS rates in these trials ranged from 28.9% to 33.3%, while ICANS rates ranged from 4.4% to 11% with both trials showing CRS and ICANS events were limited to grade 1 and 2. More importantly, outpatient BsAbs step-up dose administration can lead to increased access to care allowing community sites to be able to administer teclistamab and extend options to a broader patient population. As data continues to evolve bispecific administration and its place in therapy in the overall myeloma picture may become clearer.

## Conclusion

Accelerated dose escalation of teclistamab yielded safety event rates comparable to those in the literature. This method can optimize hospitalization periods and streamline patient care. Additional data is required to determine safety event rates between accelerated versus regular dosing.
